# Mechanisms Underlying Neuroprotection by the NSAID Mefenamic Acid in an Experimental Model of Stroke

**DOI:** 10.3389/fnins.2019.00064

**Published:** 2019-02-07

**Authors:** Parto S. Khansari, Robert F. Halliwell

**Affiliations:** ^1^School of Pharmacy and Pharmaceutical Sciences, Stony Brook University, Stony Brook, NY, United States; ^2^Thomas J. Long School of Pharmacy and Health Sciences, University of the Pacific, Stockton, CA, United States

**Keywords:** ischemic stroke, MCAO, excitotoxicity, neuroprotection, fenamates

## Abstract

Stroke is a devastating neurological event with limited treatment opportunities. Recent advances in understanding the underlying pathogenesis of cerebral ischemia support the involvement of multiple biochemical pathways in the development of the ischemic damage. Fenamates are classical non-steroidal anti-inflammatory drugs but they are also highly subunit-selective modulators of GABA_A_ receptors, activators of I_KS_ potassium channels and antagonists of non-selective cation channels and the NLRP3 inflammosome. In the present study we investigated the effect of mefenamic acid (MFA) in a rodent model of ischemic stroke and then addressed the underlying pharmacological mechanisms *in vitro* for its actions *in vivo*. The efficacy of MFA in reducing ischemic damage was evaluated in adult male Wistar rats subjected to a 2-h middle cerebral artery occlusion. Intracerebroventricular (ICV) infusion of MFA (0.5 or 1 mg/kg) for 24 h, significantly reduced the infarct volume and the total ischemic brain damage. *In vitro*, the fenamates, MFA, meclofenamic acid, niflumic acid, and flufenamic acid each reduced glutamate-evoked excitotoxicity in cultured embryonic rat hippocampal neurons supporting the idea that this is a drug class action. In contrast the non-fenamate NSAIDs, ibuprofen and indomethacin did not reduce excitotoxicity *in vitro* indicating that neuroprotection by MFA was not dependent upon anti-inflammatory actions. Co-application of MFA (100 μM) with either of the GABA_A_ antagonists picrotoxin (100 μM) or bicuculline (10 μM) or the potassium channel blocker tetraethylammonium (30 mM) did not prevent neuroprotection with MFA, suggesting that the actions of MFA also do not depend on GABA_A_ receptor modulation or potassium channel activation. These new findings indicate that fenamates may be valuable in the adjunctive treatment of ischemic stroke.

## Introduction

Ischemic stroke is a major cause of human neurological morbidity and mortality and is associated with a poor prognosis around the world (Maiese, 1998; Beresford et al., 2003). According to an update by the American Heart Association, stroke remains the fourth leading cause of death in United States with close to 800,000 new and recurrent cases annually (Benjamin et al., 2018). Significantly, a lack of efficacy in clinical trials of the drugs directed at a single pharmacological or biochemical target has raised the hypothesis that treatment of ischemic stroke may require pharmacological interventions that can modulate a number of activated pathways (Sweeney et al., 1995; Hammerman and Kaplan, 1998; Lo et al., 2003).

Fenamates are classical non-steroidal anti-inflammatory drugs that inhibit cyclooxygenases (Asanuma et al., 2001) but additionally and possibly uniquely, they have a number of significant other pharmacological actions relevant to the pathogenesis of ischemic stroke. For example, several clinically approved and widely used fenamates are effective and selective inhibitors of the NLRP3 inflammasome via inhibition of the volume-regulated anion channel in macrophages (Daniels et al., 2016; Mangan et al., 2018). The fenamate meclofenamic acid acts as an opener of Kv7.2/3 (KCNQ2/3) potassium channels expressed in CHO cells; it enhances the M-current and hyperpolarizes membrane potential and reduces action potentials in cultured neurons (Peretz et al., 2005, 2007). The fenamate, mefenamic acid (MFA) (*Ponstel*) is a highly subunit-specific modulator of GABA currents, potentiating α1β2γ2 GABA receptors but inhibiting α1β1γ2 GABA receptors expressed in HEK293 cells or Xenopus oocytes (Halliwell et al., 1999). Moreover, MFA potentiates and directly activates GABA-A chloride currents in cultured rodent hippocampal neurons (Coyne et al., 2007). In addition, niflumic and flufenamic acid, inhibit NMDA-activated currents recorded from spinal neurons (Lerma and Martin del Rio, 1992) and flufenamic acid has been shown to block calcium activated non-selective cation (CAN) channels in rat hippocampal CA1 neurons (Partridge and Valenzuela, 2000).

This complex and diverse pharmacology has led to evaluation of MFA in seizure control, pain syndromes, premenstrual syndrome, asthma, Alzheimer’s disease and even alcohol induced cognitive impairment in Zebrafish (Cimolai, 2013; Daniels et al., 2016; Rajesh et al., 2018). A previous study from this lab has indicated that systemic administration of MFA reduced brain damage in rodents *in vivo* and was neuroprotective against glutamate evoked excitotoxicity in rat hippocampal neurons *in vitro* (Khansari and Halliwell, 2009). The aims of the present study were therefore twofold: first, to determine the intrinsic efficacy of MFA to protect against transient cerebral ischemia by its direct infusion into the brain using an *in vivo* model of stroke and, secondly, to investigate the mechanism(s) underlying neuroprotection against glutamate-induced excitotoxicity *in vitro*.

## Materials and Methods

### Animal Model of Ischemic Stroke

**FIGURE 1:**
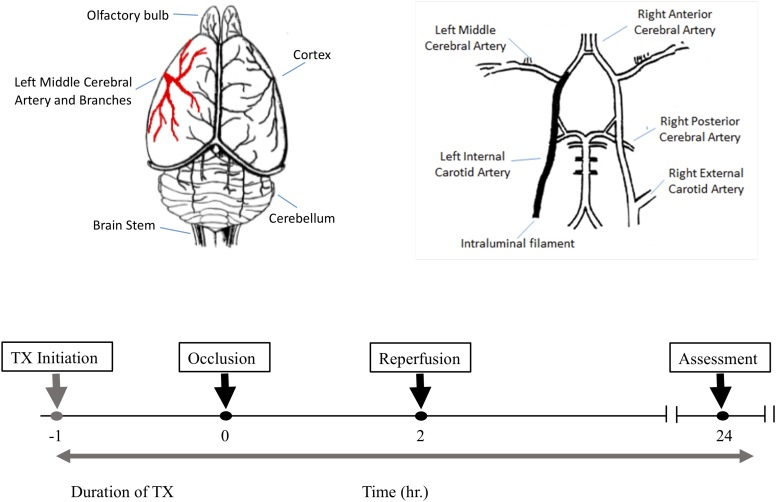
The experimental protocol and timeline for the middle cerebral artery occlusion (MCAO) experiments. Top left is a schematic dorsal view of rat brain and middle cerebral artery and its major branches supplying blood to the temporoparietal cortex. The top right illustrates a ventral view of the intraluminal filament occluding the left internal carotid artery beyond the bifurcation of the left middle cerebral artery to induce MCAO. The lower panel shows the timeline for drug treatment, MCAO, reperfusion and post-MCAO evaluation.

All animal studies conformed to the guidelines outlined in the Guide for the Care and Use of Laboratory Animals from the National Institutes of Health and were approved by the Galileo Pharmaceuticals (Santa Clara, CA, United States) Animal Use Committee. 32 male Wistar rats weighing 300–350 g were randomly assigned to one of four treatment groups (*n* = 8 per group). All animals were subjected to 2 h middle cerebral artery occlusion (MCAO) using a modified intraluminal filament technique (Figure 1). In brief, external, internal and common carotid arteries were exposed through a midline incision on the neck. A filament (Stren, Wilmington, DE, United States), its tip rounded by heating, was inserted into the right common carotid artery and advanced into the internal carotid artery to occlude the origin of the middle cerebral artery. The filament was then secured in place, the wound closed and the animals were allowed to awaken. One hour after the induction of ischemia, animals were assessed for functional impairment using the modified Bederson grading system to verify accurate occlusion of the middle cerebral artery: reduction of blood flow in this artery is closely correlated with subsequent neurological impairment. Only animals that demonstrated forelimb flexion, resistance to lateral push and spontaneous contralateral circling were included in the study. Animals that did not exhibit these behavioral changes were excluded from the study because they may only have a partial MCA occlusion. All neurological evaluations were performed by an investigator blinded to the treatment regimen. Two hours post-occlusion, the animals were re-anesthetized to remove the filament and to allow reperfusion for 24 h. Normothermia was maintained at 37.5 ± 1°C for 6 h post-MCA occlusion via external heating and cooling devices.

### Intracerebroventricular Infusion

For targeted delivery to the brain, drugs were infused into the left lateral ventricle using a 1 day (2001D) osmotic mini-pump (Alza Corporation, Palo Alto, CA, United States). 24-h osmotic pumps were filled for delivery according to the manufacturer’s guidelines. Animals were anesthetized using 3.0% isoflurane, in 30% oxygen and then stabilized on a stereotaxic device (Harvard Bioscience, Inc., Ann Arbor, MI, United States). A midline sagittal incision about 3 cm long was made just behind the eyes to expose the skull. Using bregma as the point of reference (0 mm), the following coordinates were marked on the skull: 1.5 mm lateral and 2 mm posterior to the left of bregma (Jho et al., 2003). Using a dental bur, a small hole was gently made in the skull at the designated coordinates to expose the underlying meninges. A brain infusion cannula (ALZET CO. Palo Alto, CA, United States) connected to the pump was inserted 4 mm deep from the surface of the brain. The length of the insertion was adjusted using the spacers provided by the manufacturer. The infusion cannula was then secured on the skull using dental cement adhesive. To ensure the cannula was secured in place and to prevent skin irritation caused by the dental cement, a thin layer of bone wax was placed over the skull. Thereafter, a pocket was made on the back of the animals’ neck using small hemostats to accommodate the osmotic mini pump. The pump was then connected to the brain-dwelling cannula *via* a catheter for continuous infusion over 24 h and delivery at 8 μL/h.

### Evaluation of Ischemic Damage

**FIGURE 2:**
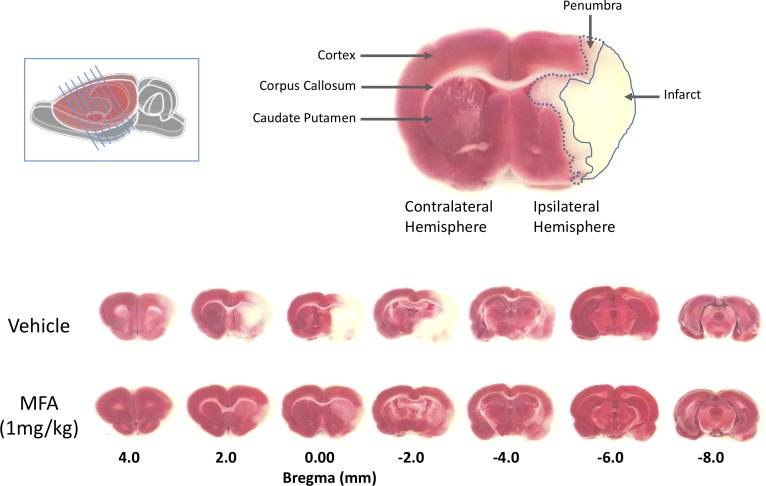
Mefenamic acid (MFA) reduces brain damage following MCAO. Left inset shows a schematic of the rat brain with the diagonal lines representing the seven 2 mm thick coronal brain sections obtained from each animal. The top right shows a coronal section 0.0 mm from bregma in a control MCAO animal. The contralateral (non-injured) hemisphere appears red due to the reaction of viable cells with the TTC stain (red). The infarct, in the core of the ipsilateral (ischemic) hemisphere in the temporal cortex, appears white due to the absence of live cells. The penumbra in the striatum appears pink due to a mixed population of both live and dead cells. The lower panels show TTC stained serial sections (rostral to caudal is left to right) from a control (vehicle) and MFA (1 mg/kg) treated animal. Note the large infarcted cortical areas in the vehicle treated animal.

Twenty-four hours post-MCAO, animals were sacrificed by CO_2_ asphyxiation. The brain was removed quickly, and seven 2-mm-thick coronal slices were cut from each brain using razor blades. The slices were immersed in a physiological solution containing 1.0% (w/v) 2,3,5-triphenyltetrazolium hydrochloride (Sigma Chemical, Co., St. Louis, MO, United States) and incubated in a 37°C water bath for 15 min. A digital photograph of each brain section was taken and used for data acquisition and analysis using the MetaMorph Imaging System and analysis software (Molecular Devices Corporation, San Jose, CA, United States). The regions of analysis were the penumbra, infarct, edema and the total ischemic brain damage (the sum of infarct and penumbra) and compensated for ipsilateral swelling of the brain as previously described (Khansari and Halliwell, 2009). The distinction between the core damage (infarct) and the compromised region (penumbra) was assessed using an automated color threshold function of the MetaMorph imaging system, by which normal brain tissue appears red, infarct region white, and penumbra as an intermediate pink surrounding the infarct region (Figure 2, top). All the measurements were obtained by an investigator blinded to the animal’s treatment group.

### Statistical Analysis

The minimum number of animals per group was calculated using the SISA program (Quantitative Skills) for population size. Sample size calculations assumed 95% power and a significance level at *p* < 0.05. All group data are presented as means ± SEM. A between-groups comparison of each region of interest was carried out using an unpaired Student’s *t*-test (between two groups) or a one-way ANOVA followed by *post hoc* Dunnett’s multiple comparisons for groups larger than two (GraphPad Prism, La Jolla, CA, United States).

### Isolation and Culturing of Primary Hippocampal Neurons

All animal studies conformed to the guidelines outlined in the Guide for the Care and Use of Laboratory Animals from the National Institutes of Health and were approved by the University of Pacific Institutional Animal Care and Use Committee. Hippocampal neurons were isolated from 19 days gestation embryos (Simonsen, Gilroy, CA, United States) as described previously (Halliwell et al., 2002) and plated on poly-D-lysine-coated 24-well plates in Neurobasal medium supplemented with B27 (1%, v/v) (Invitrogen, Carlsbad, CA, United States), Penicillin/Streptomycin (1%, v/v), and 0.5 mM L-glutamine (Sigma-Aldrich). Cells were placed in an incubator (Isotemp, Fisher Scientific, Inc., Santa Clara, CA, United States) at 37°C, 100% humidity and 5% CO_2_. Non-neuronal cell division was halted by the addition of 10 μM cytosine arabinoside (Sigma-Aldrich) after 2 days in culture. Fresh media was added every 3 days thereafter.

### Glutamate-Evoked Neurotoxicity

Neurotoxicity was induced by exposure of the neuronal cell cultures to mono-sodium glutamate (Na-glutamate). Briefly, hippocampal neurons, 9 days in culture, were exposed to 10 min of 5 μM filter-sterilized Na-glutamate. Plates exposed to glutamate were co-incubated with test compounds and placed in a humidified incubator at 37°C and 5% CO_2_. To terminate glutamate exposure, cells were washed three times in modified Neurobasal media (composition: 1% B27, 1% Pen/Strep, 0.5% glutamine). Thereafter, compounds were added back to each well so that the final concentration of the drug was the same during the glutamate exposure and overnight incubation. Twenty-four hours after exposure to Na-glutamate, cell death was quantified by measuring the release of lactate dehydrogenase (LDH) in the culture media using a CytoTox-96 non-radioactive assay kit (Promega, Madison, WI, United States). Neuroprotection was calculated as the percentage reduction in cell death induced by exposure to Na-glutamate for 10 min.

## Results

### Intrinsic Efficacy of Mefenamic Acid in MCAO Model

**FIGURE 3:**
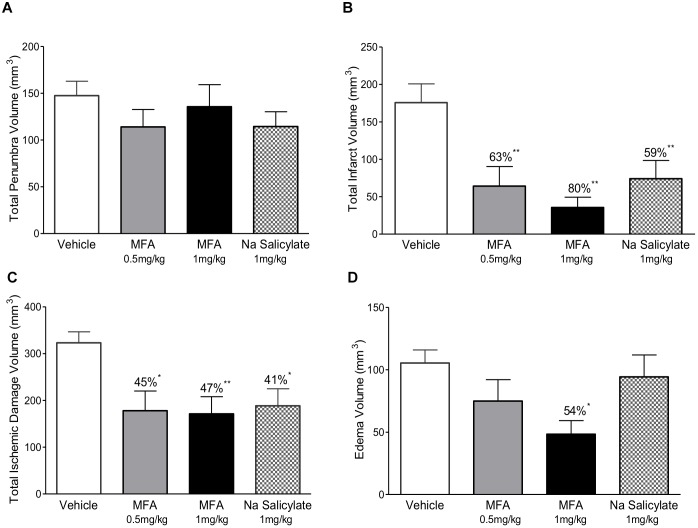
Mefenamic acid and sodium salicylate are neuroprotective in the rodent transient MCAO model. The histograms show **(A)** penumbra volume, **(B)** infarct volume, **(C)** total ischemic damage (TID) and **(D)** edema volume in animals treated with either MFA (0.5 or 1 mg/kg), sodium salicylate (1 mg/kg) or vehicle control. Drugs were infused into the left lateral cerebral ventricle by osmotic mini pump for 24 h beginning 1 h prior to MCAO. MFA significantly reduced infarct, edema, and TID; salicylate also reduced infarct and TID but the penumbra volume was not reduced in any of the treatment groups. Values are expressed as mean ± SEM for *n* = 8 in each group. ^∗^*p* ≤ 0.05; ^∗∗^*p* ≤ 0.01.

The 32 animals were randomly assigned to one of four treatment groups (with *n* = 8 per group) as follows: vehicle (NaOH, 0.1 M), MFA (0.5 mg/kg), MFA (1 mg/kg), and sodium salicylate (1 mg/kg). The dose selection was based on our pilot study (Supplementary Figure S1). Figure 1, illustrates a schematic diagram of the experimetnal protocol. One hour prior to the induction of cerebral ischemia (*t* = −1), a 24 h *Alzet* osmotic mini pump (2001D) and brain infusion cannula was implanted in the animal. As shown in Figure 2, ICV administration of MFA (1 mg/kg) reduced the ischemic damage in all seven coronal slices of the rat brain. Figure 3 summarizes that 0.5 mg/kg MFA reduced total ischemic damage (TID) by 45% (*p* < 0.05), and the infarct by 63% (*p* < 0.01), but had no significant effect on the edema volume. Similarly, treatment with 1 mg/kg of MFA decreased the TID by 47% (*p* < 0.05), and the infarct volume by 80% (*p* < 0.01). Notably edema volume was significantly reduced from 103 ± 10 mm^3^ in vehicle treated group to 48 ± 11 mm^3^ in the MFA (1 mg/kg) group; this represents a 54% (*p* < 0.05) reduction (Figure 3). Sodium Salicylate, a non-fenamate NSAID tested as a reference compound also significantly reduced the total ischemic brain damage by 42% (*p* < 0.05) and the infarct volume by 59% (*p* < 0.01), but did not reduce the edema volume. The reduction of infarct volume in MFA treated animals shows a highly significant neuroprotective effect in the MCAO model.

### Neuroprotection by Fenamates *in vitro*

**FIGURE 4:**
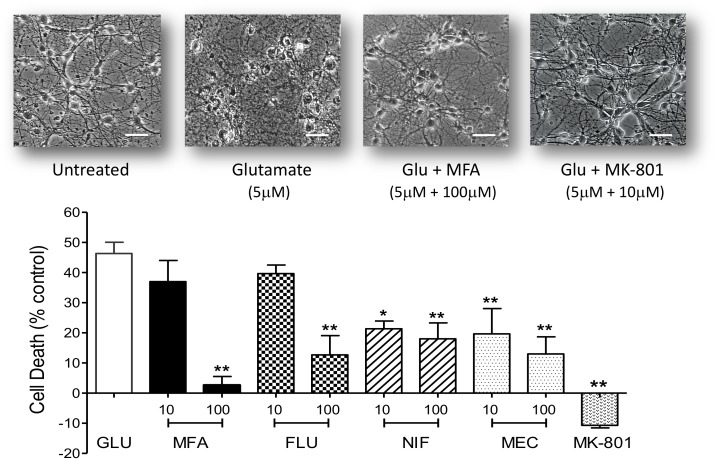
Excitotoxity was reduced by fenamate NSAIDs *in vitro*: **(top)** phase contrast photomicrographs of hippocampal neurons 9 days in culture untreated and cells after exposure to glutamate (Glu, 5 μM), glutamate plus MFA (100 μM) or glutamate plus MK-801 (10 μM). **(Bottom)** Shows histogram summaries of similar experiments conducted with mefenamic acid, flufenamic acid, niflumic acid or meclofenamic acid on glutamate induced cell death. Fenamates or MK-801 were co-incubated during glutamate exposure and immediately after exposure for 24 h. Cell death was assayed 24 h post-exposure and significantly reduced (^∗∗^*p* ≤ 0.01) by treatment with all the fenamates at 100 μM. Niflumic acid and meclofenamic acid, at 10 μM, also significantly reduced cell death by 54% (^∗^*p* ≤ 0.05) and 56% (^∗∗^*p* ≤ 0.01) respectively. Note that MK-801 even reduced LDH (cell death) below the untreated cells. The scale bar is 40 microns at 300x magnification.

Hippocampal neurons maintained in cell culture for 9 days were exposed to Na-glutamate (5 μM) for 10 min in the absence or presence of either meclofenamic acid (10 or 100 μM), niflumic acid (10 or 100 μM), flufenamic acid (10 or 100 μM) or MFA (10 or 100 μM). Doses were chosen based on the pharmacological properties of MFA (Coyne et al., 2007). As shown in Figure 4, at 100 μM all selected fenamates significantly reduced cell death *in vitro*. Treatment with MK-801 (10 μM) which we used as a reference compound also showed almost complete protection against glutamate-induced cell death. The rank order of efficacy for protection against excitotoxicity by the fenamates at 100 μM from highest was: mefenamic acid > flufenamic acid ≥ meclofenamic acid > niflumic acid. These data therefore show that neuroprotection is a property of the fenamates class of NSAID.

### Mechanism Underlying the Neuroprotective Effects of Fenamates

#### Cyclooxygenase Inhibition

**FIGURE 5:**
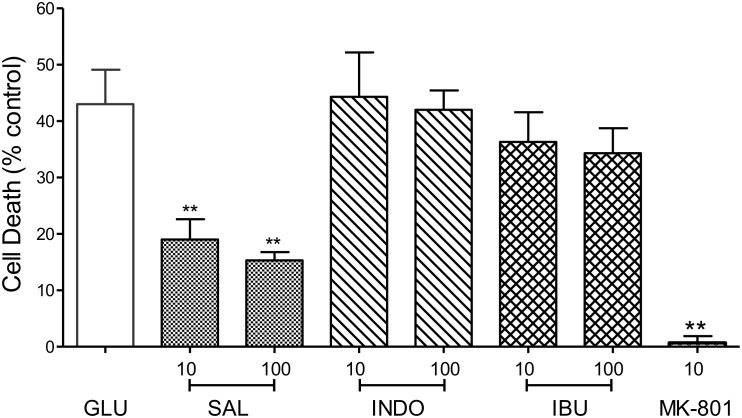
Impact of Cyclooxygenase inhibition on glutamate-evoked neurotoxicity: hippocampal neurons 9 days in culture were exposed to glutamate (5 μM) in the absence (control) or presence of either sodium salicylate (SAL), indomethacin (INDO), or ibuprofen (IBU) (each at 10 and 100 μM). Drugs were co-incubated during glutamate exposure and immediately after exposure for 24 h. Cell death was measured 24 h post-exposure using Cytotox-96. Sodium salicylate significantly reduced cell death by 56% (^∗∗^*p* ≤ 0.01) and 65% (^∗∗^*p* ≤ 0.01) at 10 and 100 μM, respectively. However, neither indomethacin nor ibuprofen reduced glutamate-induced cell death. The NMDA channel blocker, MK-801 (10 μM) reduced cell death by 98% (^∗∗^*p* ≤ 0.01).

To address the hypothesis that the neuroprotective effects of MFA and other fenamates results from COX inhibition, indomethacin (10 or 100 μM), ibuprofen (10 or 100 μM), and sodium salicylate (10 or 100 μM), were examined in our glutamate-induced neurotoxicity assay. In keeping with initial observations, cell death was reduced by 79% (*p* < 0.01) and 65% (*p* < 0.01) by MFA (100 μM) and sodium salicylate (100 μM), respectively (Figure 5). However, the NSAIDs, ibuprofen and indomethacin did not reduce glutamate evoked cell death, suggesting that COX inhibition alone is not sufficient to inhibit excitotoxicity.

#### GABA Receptor Modulation

Positive allosteric modulators of the GABA_A_ receptor are neuroprotective in several experimental models of stroke (e.g., Fan et al., 2015) and a number of fenamates potentiate neuronal GABA_A_ receptors (Halliwell et al., 1999; Coyne et al., 2007). Therefore, we tested the effects of MFA on glutamate-evoked cell death in the presence of the GABA_A_ channel blocker picrotoxin, or the competitive GABA_A_ receptor antagonist bicuculline (Johnston, 2013) to evaluate the involvement of GABA_A_ receptors in neuroprotection (see Nelson et al., 2001). In addition, the effects of two well-established potentiators of GABA_A_ receptors, i.e., the intravenous general anesthetic sodium pentobarbital, and the sedative-hypnotic benzodiazepine chlordiazepoxide, were also evaluated against glutamate-evoked cell death.

**FIGURE 6:**
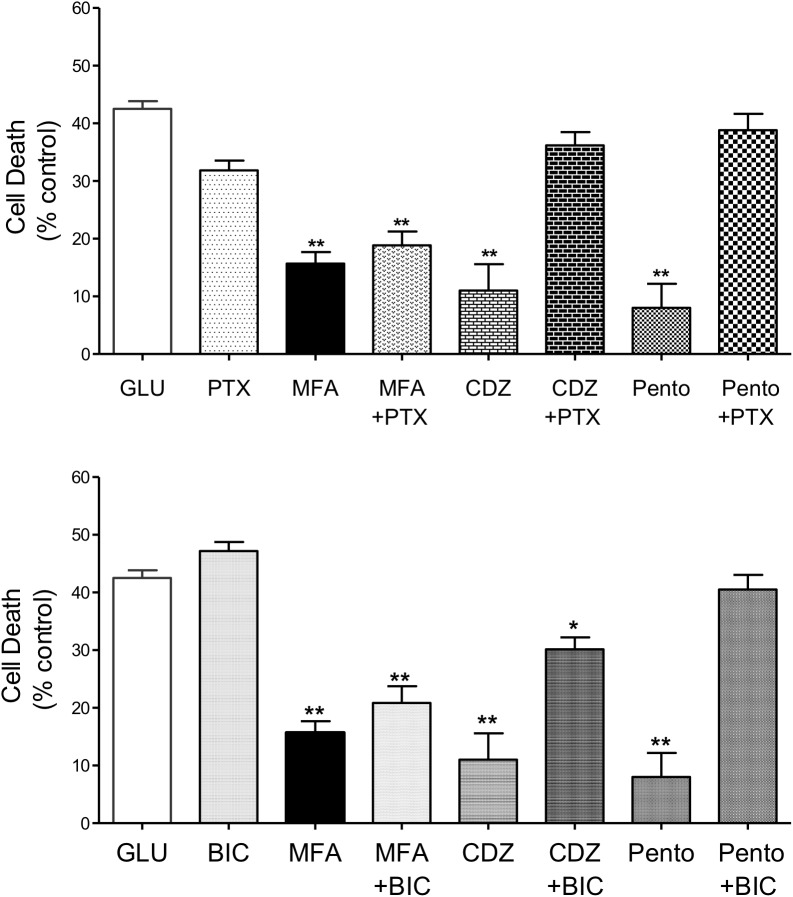
Impact of GABA_A_ receptor and ion channel inhibition on the neuroprotective effects of MFA *in vitro*: hippocampal neurons cultured for 9 days were exposed to glutamate (5 μM) and test drugs by co-incubating during exposure and immediately after exposure for 24 h. Cell death was quantified using Cytotox-96 at 24 h post-exposure. **(Top)** Treatment with MFA (100 μM), chlordiazepoxide (CDZ, 100 μM), or sodium pentobarbital (Pento, 100 μM) reduced glutamate induced cell death by 62, 74, and 81% (^∗∗^*p* ≤ 0.01) respectively. Picrotoxin (PTX, 100 μM) abolished the neuroprotective effect of CDZ and sodium pentobarbital on glutamate-evoked cell death but did not significantly alter the action of MFA. Picrotoxin (100 μM) alone did not reduce glutamate evoked cell death. **(Bottom)** MFA, CDZ or pentobarbital (all tested at 100 μM) reduced glutamate induced cell death by 62, 74, and 81% (^∗∗^*p* ≤ 0.01) respectively. Bicuculline (BIC, 10 μM) abolished the effect of pentobarbital and significantly reduced the neuroprotective effect of CDZ on glutamate induced cell death, but failed to alter the effect of MFA. Bicuculline (10 μM) alone did not reduce glutamate evoked cell death ^∗^*p* < 0.05.

Co-application of MFA (100 μM) with picrotoxin (100 μM) did not significantly change the neuroprotective effects of MFA (100 μM). In stark contrast, reduction in glutamate-induced cell death by chlordiazepoxide (100 μM) or pentobarbital (100 μM) was abolished by the co-application of picrotoxin (100 μM) whereas picrotoxin itself had no effect on glutamate-induced cell death (Figure 6, top). Consistent with these findings, bicuculline (10 μM) did not change the level of cell death induced by glutamate and co-incubation of bicuculline (10 μM) with MFA did not significantly change the neuroprotective effect of MFA alone (Figure 6, bottom). In contrast, bicuculline (10 μM) significantly reduced the neuroprotective effects of both chlordiazepoxide (100 μM) and pentobarbital (100 μM) against glutamate excitoxicity. Collectively, these data show that the ability of MFA to reduce glutamate-evoked cell death is not dependent on GABA_A_ receptor modulation.

#### Potassium Channel Activation

Nicorandil, a non-selective K_ATP_ channel opener is neuroprotective against glutamate-induced cell death (Nagy et al., 2004). Notably, MFA is also reported to activate K_ATP_ channels (Teramoto et al., 2003). Therefore, we addressed whether potassium channel activation might be an underlying mechanism for the neuroprotection observed with MFA. To inhibit potassium channels, tetraethylammonium (TEA) (Heurteaux et al., 1993), a broad spectrum potassium channel blocker, which inhibits nicorandil induced outward currents (Kajioka et al., 1990) was tested on the actions of MFA and on the effects of nicorandil in our excitotoxicity assay.

**FIGURE 7:**
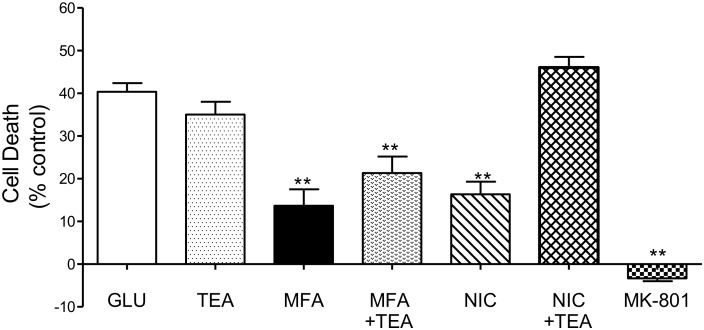
Involvement of K channels in the neuroprotective effects of MFA *in vitro*: hippocampal neurons in culture for 9 days were exposed to glutamate (5 μM). Drugs were co-incubated during glutamate exposure and immediately after for 24 h. Cell death was quantified using the Cytotox-96 24 h later. Treatment with MFA (100 μM) or nicorandil (NIC, 100 μM) reduced glutamate mediated cell death by 65 and 60% (^∗∗^*p* ≤ 0.01) respectively. Tetraethylammonium (TEA, 30 mM) alone did not significantly alter the level of glutamate induced cell death. Co-application of TEA (30 mM) completely abolished the neuroprotective effect of nicorandil (100 μM) but co-incubation of TEA (30 mM) with MFA (100 μM) did not significantly affect the neuroprotective actions of this fenamate NSAID.

Consistent with our previous experiment and as shown in Figure 7, cell death was reduced when cultures were incubated with MFA (100 μM) or nicorandil (100 μM) by 65% (*p* ≤ 0.01) and 60% (*p* ≤ 0.01), respectively. Treatment with TEA (30 mM) alone however did not significantly reduce cell death when compared with cultures exposed to glutamate. *Post hoc* analysis showed that co-application of MFA (100 μM) and TEA (30 mM) had little or no effect on the neuroprotective property of MFA. In contrast, the effect of nicorandil (100 μM) on glutamate-evoked cell death was abolished by TEA (30 mM).

## Discussion

The present study was undertaken to investigate the hypothesis that fenamate NSAIDs have intrinsic neuroprotective properties against ischemic stroke *in vivo* and to establish the underlying pharmacological mechanisms *in vitro*. Our hypothesis was based on observations that (i) fenamates have a unique spectrum of pharmacological actions that may be important in the pathogenesis of stroke and (ii) a previous report that MFA administered intravenously reduces ischemic brain damage induced by transient ischemic stroke in the rodent MCAO model of stroke (Khansari and Halliwell, 2009).

To evaluate the hypothesis that MFA efficacy is due to a direct CNS effect, rather than a peripheral and indirect site of action, we used an intracerebroventricular (ICV) route of drug delivery to the CNS. A continuous infusion for 24 h at a constant rate also enhances the availability of the drug during the course of injury when its action is likely most needed and the intraluminal filament model of stroke is a well-established and validated animal model to study neuroprotection (Herson and Traystman, 2014). MFA administration significantly reduced the total ischemic brain damage and the infarct volume to an extent normally only achieved with a potent neuroprotectant like MK-801 (*Dizilopine^®^*). Notably, edema volume was also reduced significantly in MFA treated animals (but not in the sodium salicylate treated group). These data are the first demonstration of neuroprotection *in vivo* against MCAO with direct infusion of the NSAID, MFA.

*In vitro*, four fenamate NSAIDs were tested against glutamate induced excitotoxicity. Treatment with MFA, flufenamic acid, niflumic acid or meclofenamic acid all significantly reduced cell death. Consistent with our observations, Chen and colleagues, have shown that exposure of the chick retina *in vitro* to oxygen-glucose deprivation can be attenuated by mefenamic acid, flufenamic acid, and meclofenamic acid (Chen et al., 1998). In addition, these authors also reported a neuroprotective effect of fenamates against the direct application of NMDA or Kainate to the retinal slices. Together, these findings show that fenamate NSAIDs have significant neuroprotective properties against excitotoxicity induced cell death.

The neuroprotective effects of MFA and other fenamates may result from a single or a combination of several mechanisms. To address the importance of each of the potential mechanisms against glutamate neurotoxicity, a prototype agent which shares a specific component of the fenamates activity was evaluated in hippocampal neuron cell cultures. Inhibition of cyclooxygenases by fenamates is well-documented. We therefore compared the neuroprotective effects of MFA with two potent non-selective COX inhibitors, indomethacin and ibuprofen and found they had no effect on glutamate-induced cell death. In contrast, salicylate reduced cell death. Consistent with our findings, De Cristóbal et al. (2002), reported that indomethacin did not protect rat cortical neurons in culture against oxygen-glucose deprivation whereas aspirin and sodium salicylate were neuroprotective (De Cristóbal et al., 2002). Taken together, the lack of neuroprotection by indomethacin and ibuprofen, and the wide spectrum of pharmacological properties of both MFA and sodium salicylate suggest that attenuation of glutamate induced cell death may result from actions other than COX I and II inhibition.

Modulation of GABA_A_ receptors has been associated with neuroprotection in several experimental models of cerebral ischemia (e.g., Håberg et al., 2001) and fenamates have been shown to modulate GABA_A_ receptors in electrophysiological studies of primary neurons and cells expressing recombinant human GABA_A_ receptors (Halliwell et al., 1999; Coyne et al., 2007). Here we show that pentobarbital and chlordiazepoxide greatly reduced neuronal cell death. Rekling (2003) previously reported that phenobarbital and chlordiazepoxide inhibited oxygen-glucose deprivation induced cell death in rat hippocampal slices. These data are consistent with the hypothesis that potentiation of GABA_A_ receptors could be an important underlying mechanism against excitotoxicity (Foster and Kemp, 2006; Amantea and Bagetta, 2017). However, co-incubation of MFA with either picrotoxin, a non-competitive GABA gated chloride channel blocker (Squires et al., 1983) or the competitive GABA_A_ receptor antagonist, bicuculline (Johnston, 2013), had no effect on the efficacy of MFA to inhibit glutamate-induced cell death. In stark contrast, reductions in glutamate cell death by pentobarbital and chlordiazepoxide were completely reversed by both GABA_A_ antagonists. Our observations, together with the finding that niflumic acid, which inhibits GABA_A_ receptors (Sinkkonen et al., 2003), indicate that fenamate neuroprotection does not depend on modulation of GABA-gated chloride channels.

Some evidence suggests that the opening of potassium channels leads to efflux of potassium ions and counteracts the depolarizing effect of ischemic insult (Heurteaux et al., 1993; Veltkamp et al., 1998). Several types of potassium channels have been identified in the brain, including ATP-sensitive potassium channels present in the cerebrovasculature and the hippocampus (Rosenblum, 2003). Teramoto et al. (2003) reported that a unique property of MFA is activation of both ATP-sensitive potassium channels and intracellular calcium activated potassium channels. We therefore addressed the effects of the potassium channel opener, nicorandil (SG-209), a K_ATP_ channel opener (Levin et al., 2001) on glutamate-evoked cell death and found it profoundly reduced glutamate induced cell death. Thus, to investigate the role of potassium channel activation by MFA in reducing glutamate-induced cell death, TEA, a broad spectrum potassium selective ion channel blocker was co-incubated with MFA or nicorandil. Our results showed that TEA completely abolished the effect of nicorandil against glutamate evoked cell death but had little or no effect on the neuroprotective actions of MFA. These data therefore support the hypothesis that a variety of potassium channels activators might be useful neuroprotective agents (Heurteaux et al., 1993; Hewawasam et al., 2003), but this property of MFA is not likely to be the mechanism for its neuroprotective actions.

Mefenamic acid has been shown to reduce cell death induced by amyloid β_1–42_ in neurally differentiated PC-12 cells *in vitro* and to attenuate the cognitive impairments of amyloid β_1–42_ in a rodent model of spatial learning and memory in the water maze task (Joo et al., 2006). These neuroprotective effects were suggested to result from the efficacy of MFA to inhibit the release of reactive oxygen species (ROS), nitric oxide accumulation and mitochondrial cytochrome c, and upregulation of the anti-apoptotic protein, Bcl-X_L_. Preliminary experiments in this lab also show that MFA reduces ROS released from neurons exposed to the excitotoxin glutamate (Supplementary Figure S2) but further studies are underway. In a recent study, MFA was additionally reported to inhibit the NLRP3 inflammasome (a multi-protein complex implicated in inflammatory diseases) *via* inhibition of the volume-regulated anion channel and to protect Lister hooded rats against Aβ_1–42_ induced impairments in the novel object recognition task (Daniels et al., 2016). The data from these two studies therefore indicates additional mechanisms for neuroprotection observed with fenamates against inflammation in the brain and have also suggested that fenamates might be usefully repurposed to target inflammatory disorders such as Alzheimer’s disease (Mangan et al., 2018). Further studies, however, are therefore required to delineate the precise mechanisms of action of fenamates against ischemic insult and neurodegeneration.

## Conclusion

Our own experiments show that MFA has significant efficacy to reduce brain damage from ischemic stroke *in vivo* and that fenamates, as a class, an ability to reduce neuronal cell death from excitotoxicity *in vitro*. MFA has a unique pharmacology, including an ability to inhibit cyclooxygenases, modulate GABA_A_ receptors, activate potassium channels and inhibit the inflammasome, all of which may contribute synergistically to its neuroprotective efficacy. On the basis of the pharmacological properties of fenamates, it may be of considerable value to further address their neuroprotective properties experimentally and clinically, as adjunctive therapy in the treatment of ischemic stroke.

## Author Contributions

All authors listed have made a substantial, direct and intellectual contribution to the work, and approved it for publication.

## Conflict of Interest Statement

The authors declare that the research was conducted in the absence of any commercial or financial relationships that could be construed as a potential conflict of interest.

## Supplementary Material

FIGURE S1 Pilot data showing that 1 mg/kg mefenamic acid, administered ICV reduces brain damage following MCAO. **(A)** Shows a 2 mm thick coronal brain section −1 mm from bregma in an animal given 1 mg/kg mefenamic acid (MFA) by ICV (see section “Materials and Methods” for further details). The contralateral (non-injured) hemisphere is red due to the reaction of viable cells with the TTC stain (red). The infarct, in the core of the ipsilateral (ischemic) hemisphere is white due to the absence of live cells. **(B)** Show a 2 mm thick coronal brain section (−1 mm from bregma) from an animal given 0.1 mg/kg MFA ICV. Note the large infarcted cortical areas in this brain section.

FIGURE S2 Impact of NSAIDs on reactive oxygen species (ROS) formation: ROS was assayed using 2′, 7′-dichlorofluorescein diacetate (DCFH-DA). This dye rapidly enters the cells and is desterified to the ionic dichlorofluorescein (DCFH). DCFH is oxidized to the fluorescent 2′, 7′-dichlorofluorescein (DCF) by ROS. A microplate fluorescent reader was used to measure the fluorescence at excitation and emission of 485 and 530 nm, respectively. Primary hippocampal neurons were washed twice after treatment and incubated for 15 min at 37°C with 10 μM dye in media without serum and phenol red. ROS formation was significantly increased in hippocampal cultures after exposure to glutamate (5 μM; *p* < 0.01). In contrast, ROS levels in culture media decreased significantly when treated with MFA (30 or 100 μM). ROS was also reduced in cultures treated with MK-801 (10 μM) and indomethacin (100 μM). The experiment was conducted using a single culture of hippocampal neurons with each condition ran in triplicate. ^∗^*p* ≤ 0.05; ^∗∗^*p* ≤ 0.01).
