# Supplementary figures and images for: Mechanisms Underlying Neuroprotection by the NSAID Mefenamic Acid in an Experimental Model of Stroke
Source: Front Neurosci. 2019 Feb 7;13:64. doi: 10.3389/fnins.2019.00064 (PMC6374636; doi:10.3389/fnins.2019.00064)

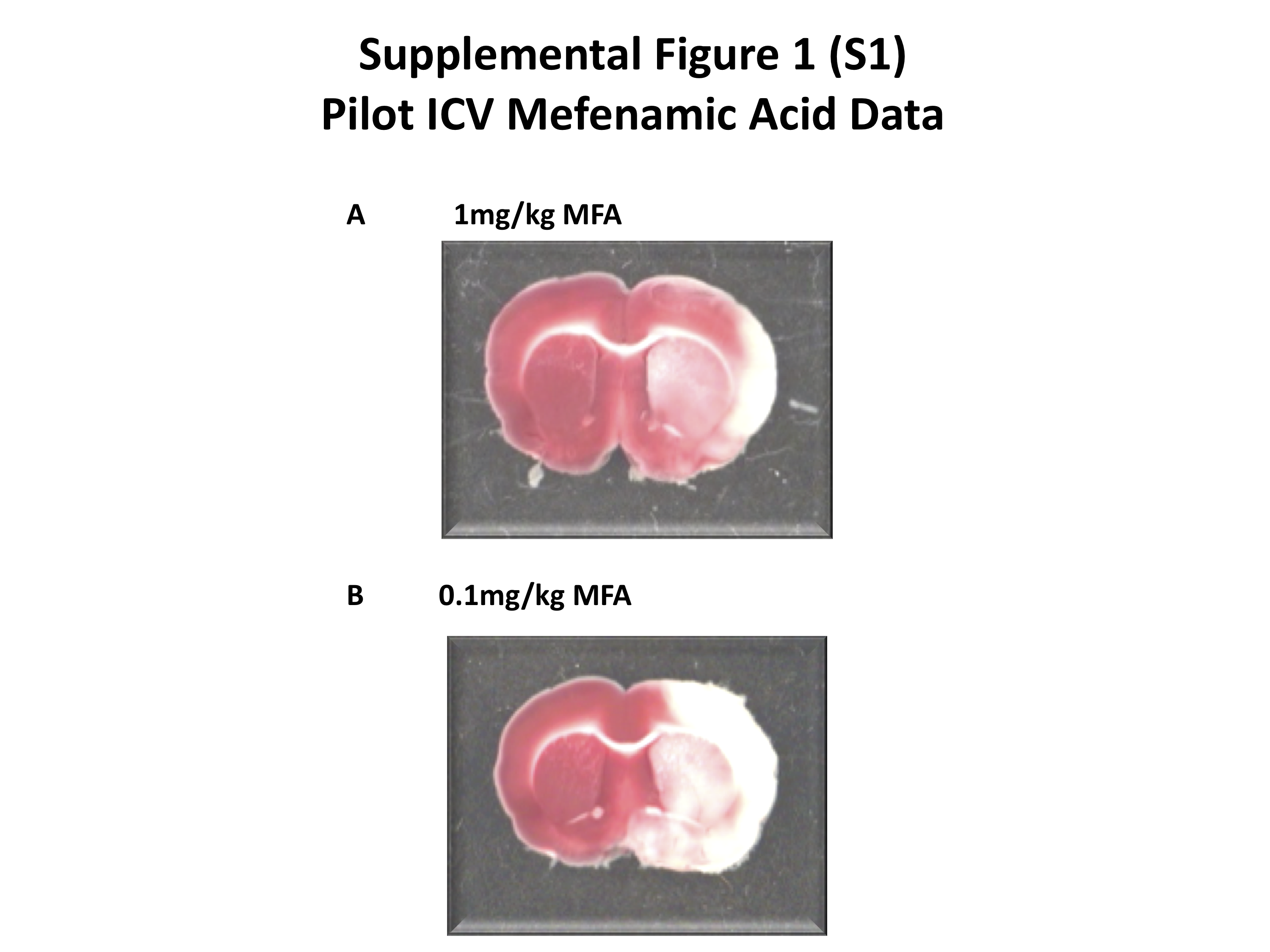

Supplement: FIGURE S1 — Pilot data showing that 1 mg/kg mefenamic acid, administered ICV reduces brain damage following MCAO. (A) Shows a 2 mm thick coronal brain section −1 mm from bregma in an animal given 1 mg/kg mefenamic acid (MFA) by ICV (see section “Materials and Methods” for further details). The contralateral (non-injured) hemisphere is red due to the reaction of viable cells with the TTC stain (red). The infarct, in the core of the ipsilateral (ischemic) hemisphere is white due to the absence of live cells. (B) Show a 2 mm thick coronal brain section (−1 mm from bregma) from an animal given 0.1 mg/kg MFA ICV. Note the large infarcted cortical areas in this brain section. [file Image_1.tiff]

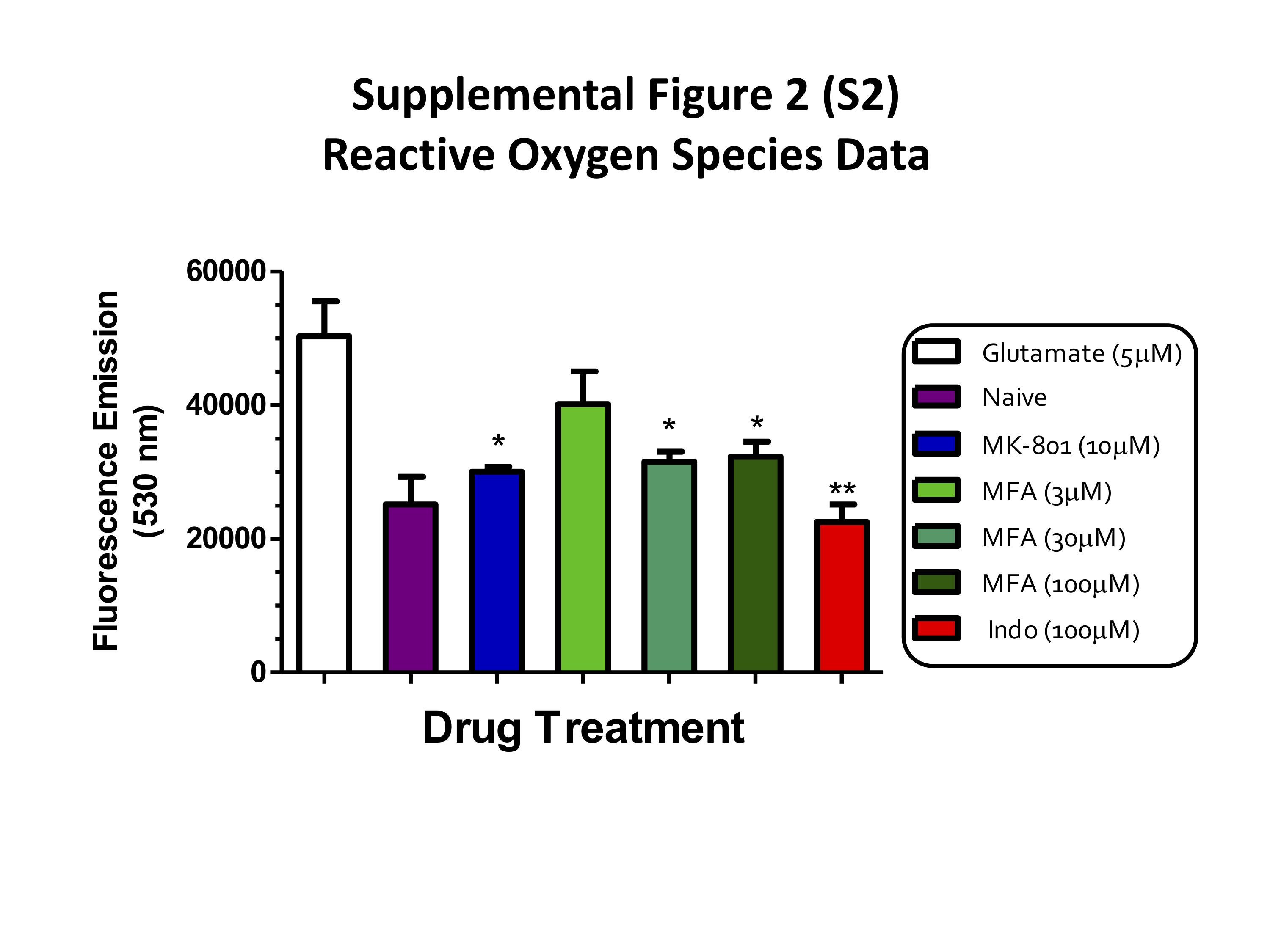

Supplement: FIGURE S2 — Impact of NSAIDs on reactive oxygen species (ROS) formation: ROS was assayed using 2′, 7′-dichlorofluorescein diacetate (DCFH-DA). This dye rapidly enters the cells and is desterified to the ionic dichlorofluorescein (DCFH). DCFH is oxidized to the fluorescent 2′, 7′-dichlorofluorescein (DCF) by ROS. A microplate fluorescent reader was used to measure the fluorescence at excitation and emission of 485 and 530 nm, respectively. Primary hippocampal neurons were washed twice after treatment and incubated for 15 min at 37°C with 10 μM dye in media without serum and phenol red. ROS formation was significantly increased in hippocampal cultures after exposure to glutamate (5 μM; p < 0.01). In contrast, ROS levels in culture media decreased significantly when treated with MFA (30 or 100 μM). ROS was also reduced in cultures treated with MK-801 (10 μM) and indomethacin (100 μM). The experiment was conducted using a single culture of hippocampal neurons with each condition ran in triplicate. ∗p ≤ 0.05; ∗∗p ≤ 0.01). [file Image_2.tiff]
